# Early recognition of coeliac disease through community pharmacies: a proof of concept study

**DOI:** 10.1007/s11096-016-0368-4

**Published:** 2016-08-08

**Authors:** Heidi Urwin, David Wright, Michael Twigg, Norma McGough

**Affiliations:** 1 School of Pharmacy, University of East Anglia, Norwich, NR4 7JT UK; 2 Coeliac UK, 3rd Floor, Apollo Centre, Desborough Avenue, High Wycombe, Bucks HP11 2QW UK

**Keywords:** Case-finding, Coeliac disease, Community pharmacy, Point of care testing, Screening, United Kingdom

## Abstract

*Setting* Fifteen community pharmacies in the UK. *Objective* Proof of concept study to test the use of community pharmacies for active case finding of patients with coeliac disease. *Methods* Customers accessing over-the counter and prescription medicines indicated in the treatment of possible symptoms of coeliac disease over a 6 month period were offered a free point of care test. All patients were given advice regarding the test results and those who tested positive were advised to make an appointment with their general practitioner. Patients and pharmacists involved in service provision were asked to complete a satisfaction survey. Pharmacists were additionally invited to undertake interviews to better understand their views on the service. *Main outcome measures* Feasibility of service, acceptability to stakeholders and proportion testing positive for coeliac disease. *Results* Of the 551 individuals tested, 52 (9.4 %) tested positive. 277 (50.3 %) were tested for accessing irritable bowel syndrome treatment, 142 (25.8 %) due to presenting for diarrhoea. The proportion of patients testing positive with different symptoms or for different treatments were similar. Of 43 customers who returned the satisfaction survey, all would recommend the service to others, believing the community pharmacy to be a suitable location. Community pharmacists believed that it enabled them to improve relationships with their customers and that medical practices were receptive to the service. *Conclusion* This proof of concept study has shown that community pharmacies using a point of care test can effectively recognise and refer patients for confirmatory coeliac disease testing with high levels of customer and service provider satisfaction.

## Impact of findings on practice

- Pharmacists and their staff should routinely explore symptoms of diarrhoea to ensure that they are not long term or recurrent as this may be indicative of undiagnosed coeliac disease.
- Patients who are receiving treatment for irritable bowel syndrome, haven’t previously been tested for coeliac disease and don’t experience symptom resolution should be referred for possibility of coeliac disease.
- Community pharmacy is seen by both patients and pharmacists as an appropriate location for the early recognition of patients for whom further tests to confirm diagnosis of coeliac disease are appropriate.

## Introduction

Coeliac disease is an autoimmune condition triggered by intolerance to gluten and characterised by inflammation of the small intestine [1]. Whilst it is believed to affect 1 % of the UK population [2, 3] only 24 % of those affected are diagnosed [4]. Consequently there is an estimated half a million people with coeliac disease in the UK who remain undiagnosed, reasons for which may include a lack of awareness of the condition and misdiagnosis. One in four people diagnosed with coeliac disease have previously been treated for irritable bowel syndrome (IBS) [5]. The symptoms of coeliac disease are similar to those of many minor gastrointestinal problems which people may prefer to discuss with their local pharmacist rather than having to make an appointment to see their general practitioner (GP) [6].

Delayed diagnosis and untreated coeliac disease is associated with unexplained infertility, osteoporosis and in rare cases, small bowel lymphoma [7]. The direct costs associated with undiagnosed coeliac disease are increased visits to the GP, use of medicines for symptomatic treatment, increased investigations and referral [8, 9]. Whilst screening and treating people with coeliac disease results in significant improvements in quality of life [10, 11] systematic population screening is not recommended in the UK [12] with current guidance recommends targeted screening of those individuals with related symptoms or associated conditions [7]. Individuals meeting the criteria for screening should be offered serological tests for both total immunoglobulin A (IgA) and IgA tissue transglutaminase antibodies (IgA tTGA). Total IgA levels are required as approx. 2–3 % of patients with coeliac disease will have an IgA deficiency [13, 14] and hence if IgA tTGA is tested in isolation it can increase the number of false negative results. Adults with a positive serological test result should always be referred to secondary care for endoscopy with biopsy to confirm or exclude coeliac disease [7].

Traditionally the identification and implementation of targeted screening has solely been the role of primary care physicians. They are however only able to identify patients who present to them for treatment and advice. It is more usual for patients with minor gastrointestinal symptoms to self-treat with therapy which is available from pharmacies and supermarkets [15]. To improve detection rates within Hungary, district nurses were successfully used in primary care to proactively screen for coeliac disease in young children using a finger prick blood test [16] thus demonstrating that other healthcare professionals located in primary care can effectively undertake screening for coeliac disease.

Community pharmacies are increasingly being used to deliver screening services due to their geographical spread, extended opening hours, availability of trained healthcare professionals and use by patients who may not normally access medical services [17–19]. Furthermore with access to patient medication records, community pharmacists can identify patients treated for conditions which may be indicative of coeliac disease. The recent availability of reliable point of care tests (POCTs) [20] enables community pharmacies to offer and undertake screening for coeliac disease.

### Aim of the study

The aim of this paper is to describe the proof of concept study for a targeted case finding service for coeliac disease using a small number of community pharmacies and determine both its feasibility as a service and acceptability to stakeholders.

## Ethics approval

The study was deemed to be a service evaluation by the University of East Anglia Faculty of Medicines and Health Sciences Ethics Committee.

## Methods

The case finding, proof of concept, service was provided between April and October 2015.

### Setting

Fifteen community pharmacies across England recruited through the National Association of Primary Care’s Primary Innovation Network, and including Rowlands Pharmacy and an Independent pharmacy agreed to participate. Pharmacies were purposively recruited to ensure a wide geographical spread, a mix of pharmacy locations i.e. linked to GP practice or stand alone and from large multiple and independently managed companies. Participating pharmacies were asked to contact local GP practices to inform them of the study taking place.

### Training and support

Participating pharmacists were expected to undertake an online module on recognising and managing coeliac disease from the Centre for Pharmacy Postgraduate Education, learning pharmacy website [21]. Posters for raising awareness and information leaflets were supplied by Coeliac UK.

Senior pharmacists from the different companies were involved in the service design and trained by Tillotts Pharma Ltd on the use of the Simtomax^®^ POCTs. These individuals then trained staff within the recruited pharmacies on the use of the POCTs, including how to identify, approach and recruit patients and provide feedback after testing. The majority of pharmacists also received in house training, direct from Tillotts Pharma Ltd.

Pharmacies received remuneration on commencement of training, half way through and at the end of the study.

### Service population

All customers who met the inclusion criteria outlined below were given an information sheet which explained the purpose of the service and process.

Inclusion criteria:

- Men and women aged 18 years and over receiving prescribed treatments, or requesting OTC treatments for either IBS and or iron, vitamin B12 or folate deficiency anaemia.
- No previous diagnosis or investigation for coeliac disease.
- Registered with a GP.

Those who subsequently expressed an interest were then given a short questionnaire to complete to enable any of the following exclusion criteria to be identified.

Exclusion criteria:

- Adults on a gluten-free diet or people excluding gluten from their diets.
- Adults with learning disabilities.
- Adults who have a terminal illness.
- Adults unable to give verbal consent.
- Women receiving folate due to pregnancy.
- Adults previously tested for coeliac disease with a negative result.

Symptoms and diagnoses of any associated conditions were recorded using categories provided within the bespoke pharmacy software which were based on NICE guidance [7] and verbal consent obtained before testing was undertaken.

Patients declining the test were asked to fill in a short questionnaire to capture their reasons for declining, provided with an information leaflet on coeliac disease, and signposted to their GP should any symptoms persist or re-appear.

### Intervention

The test, which measured both total immunoglobulin A (IgA) and IgA tissue transglutaminase antibodies (IgA tTGA), was provided for free to the patient and was undertaken in a consultation room within the pharmacy by a trained member of the pharmacy team. A finger prick blood sample was taken and inserted into the testing device as per standard operating procedure. In a pilot study the specificity and sensitivity of the POCT used were found to be consistent with current NHS laboratory tests for this disease [20].

Information provided in all patient leaflets highlighted the possibility of false positive or negative test results and outlined the need for further referral to confirm test results in line with national guidance. Leaflets were designed to ensure that participants were aware of the implications of the test result and could make fully informed decisions as to their future actions. On receipt of the result all participants were given a letter containing the results for their records and the opportunity to ask further questions.

Customers with negative results were given a patient leaflet, informed that the POCT indicated that they were unlikely to have coeliac disease but if problems persist to speak with their GP.

Customers with a positive result were provided with a description of the POCT, confirmation of the result, an information leaflet on coeliac disease, advised to see their GP and not to change their diet as a result of this screening test.

### Sample size justification

A pragmatic approach was applied and each pharmacy was requested to carry out 40 POCTs during the study period, which after allowing for training and preparation for the service in each pharmacy, equated to 2 POCTs per week.

Assuming that 600 POCTs were performed if the detection rate was similar to that previously reported in an at risk population of 9.6 % [22] then this would provide a 95 % CI of ±2.4 %.

### Evaluation

For each participant their age, gender, medication which triggered the approach for recruitment, present symptoms and outcome of the test result were recorded.

All service recipients were asked to complete a short satisfaction questionnaire which included a question on whether they would have been prepared to pay for the service and how much they would be willing to pay.

The pharmacy team also completed a baseline and end of study questionnaire to determine their experience of providing the service.

Interviews were undertaken with participating pharmacists, transcribed verbatim and anonymised by a representative from Coeliac UK. Analysis consisted of a framework approach [23]. After familiarisation with the data a coding framework was developed and subsequently applied to the transcripts. Constant reference was made to the transcripts when abstracting themes and sub-themes from the data to ensure meaning and context were retained. Interpretation of the transcripts was checked by a second researcher.

## Results

The minimum number of patients recruited in one pharmacy was 9 with the maximum being 63 and 11 pharmacies out of 15 recruited to target.

551 people were tested for coeliac disease using the POCT. Reasons for declining the test were provided by 15 individuals, 7 (46.7 %) stated lack of time as the primary reason for declining the service with 3 (20 %) preferring to discuss the issue with their GP. Participant demographics are provided in Table 1.

**Table 1:** Summary of demographics of recruited participants (n = 551)

Characteristic	Group	No. (%)
Gender	Female	340 (61.7)
Age (years)	18–30	123 (24.9)
	31–40	111 (20.1)
	41–50	109 (19.8)
	51–60	85 (15.4)
	61–70	66 (12.0 %)
	70+	42 (7.6)
	Unknown	1 (0.2)

Loperamide and mebeverine were the most common medicines reported for triggering recruitment (summarised in Table 2). 312 (56.6 %) patients were recruited due to purchasing over the counter (OTC) medicines which are potentially indicative of coeliac disease.

**Table 2:** Indications for medicines which triggered recruitment of participants (n = 551)

Condition	Trigger no. (%)		Total no. (%)
	Over the counter	Prescription	
Irritable bowel disease	152 (48.7)	125 (52.3)	277 (50.3)
Diarrhoea	122 (39.1)	20 (8.4)	142 (25.8)
Anaemia	16 (5.1)	58 (24.3)	74 (13.4)
Indigestion	17 (5.4)	17 (7.1)	34 (6.2)
Constipation	4 (1.3)	12 (5.0)	16 (2.9)
Other	1 (0.3)	7 (2.9)	8 (1.5)

The symptoms patients reported to be experiencing prior to consultation are provided in Table 3.

**Table 3:** Symptoms reported by participants during pharmacist consultation (n = 551)

Symptom experienced	No. (%)^a^
Regular diarrhoea	199 (36.1)
General gastro-intestinal problems	241 (43.7)
Abdominal problems	286 (51.9)
Sudden or unexpected weight loss	19 (3.4)
Regular and severe mouth ulcers	19 (3.4)
Prolonged fatigue	146 (26.5)
Regular and unexplained anaemias	61 (11.1)

^a^Participants frequently reported more than one symptom and therefore column does not add up to 100 %

Of the 551 people tested, 9.4 % (52) were given a positive result for coeliac disease. The reported symptoms and indications for treatments related to those patients who tested positive are provided in Table 4.

**Table 4:** Proportion of patients reporting different symptoms who tested positive

Reported symptom	No. (%)^a^	Indication for therapy	No. (%)^a^
Regular diarrhoea	26 (13.1)	Irritable bowel syndrome	28 (10.1)
General gastro-intestinal problems	34 (14.1)	Diarrhoea	10 (7.0)
Abdominal problems	31 (10.8)	Anaemia	7 (5.9)
Sudden or unexpected weight loss	3 (15.8)	Indigestion	2 (5.9)
Regular or severe mouth ulcers	1 (5.3)	Constipation	4 (25.0)
Prolonged fatigue	18 (12.3)		
Regular and unexplained anaemias	7 (11.5)		

^a^Based on percentage of screened patients who reported that symptom during consultation (Table 3) or indication for their therapy (Table 2)

### Customer satisfaction

Forty-three (7.9 %) customer experience questionnaires were returned. 34 (79.1 %) were from females and 4 (9.3 %) had a positive result for coeliac disease. 41 patients reported their age, 6 (14.6 %) were under the age of 30, 11 (26.8 %) from 31 to 50, 17 (41.5 %) from 51 to 70 and 7 (17.0 %) over the age of 70.

All 43 (100 %) respondents agreed that the pharmacy provided a safe and confidential environment for the service, the pharmacy team were able to answer all questions, community pharmacy was the ideal place for this type of service and would recommend the service to others.

One (2.3 %) respondent believed that service was of some value, 21 (48.8 %) believed that it was valuable and 21 (48.8 %) very valuable.

Nine (20.9 %) patients were unwilling to pay for the service, 29 (67.4 %) were willing to pay £10, 4 (9.3 %) £20 and 1 (2.3 %) £30.

A common theme was the professionalism of the service and its informative nature.

“The pharmacist was very professional” R4.

“Very pleased to know one way or another” R23.

All 12 community pharmacists who completed the survey believed that community pharmacy was a suitable location to carry out POCTs, they were confident in performing the test, were willing to continue with the service and recommend it to other pharmacists.

Eight participating pharmacists consented to be interviewed. The analysis centred on providing the service, patient feedback, interactions with GPs and future recommendations.

In terms of providing the service the pharmacists highlighted that the relatively short training session was easy to understand and effective at learning more about the condition and testing methods. Pharmacists identified that their staff could be heavily involved in identifying and testing patients from training through to service provision. One pharmacist stated that this proved useful as someone else could perform the test and then they could just look at the result and discuss this with the patient.

Pharmacists also stated this service was useful at identifying not only patients who were ‘regulars’ within their pharmacy but also those who came into buy OTC medication.

“…they were people that had come into buy something and we’ve offered them the test because of the product they were buying, looked at their symptoms.” Ph3.

Once in the consultation, the pharmacists highlighted that the service itself was relatively quick to perform and could be used as a link to other pharmacy services such as medicine use reviews (MURs).

“…when I have a patient having a test I can offer another service while they wait, so, ‘what about an MUR?’” Ph6.

Pharmacists also identified that the service helped to build rapport with patients.

“It’s definitely I think built a better rapport with them. Because I think you actually spending time with them and doing these clinical things with them I suppose builds a bit of trust with them…” Ph8.

Speed and accessibility of the service was a key advantage. Pharmacists reported that patients would often have to wait weeks or months for a test and sometimes may not be offered one by their GP. An additional perceived benefit of this quick turnaround, centred on patient reassurance.

“Well, they, to be honest a lot of them would say ‘ok, at least it’s not that” Ph4.

Finally, after discussions with both GPs and patients, pharmacists stated that GPs and medical practice staff were receptive to the service being provided in community pharmacy.

## Conclusion

Point of care testing for coeliac disease in community pharmacies was undertaken in this proof of concept study from a range of settings with the majority of pharmacies recruiting to or above expected target in the time allowed. The majority of patients who were screened were in the target age range with more than half identified because they had presented to purchase OTC products. The medicine groups which were the most likely to result in recruitment were either for the treatment of IBS or diarrhoea. Most recruits reported some form of gastrointestinal symptom with almost a quarter reporting fatigue.

Whilst point of care testing of this nature is not supported by National Institute of Health and Care Excellence (NICE) guidance, the test used mirrored NICE recommendations with total immunoglobulin A (IgA) and IgA tissue transglutaminase antibodies (IgA tTGA) both determined. The detection rate was in line with similar research which had focussed on those individuals at high risk and consequently was relatively efficient. Patients were very positive about the provision of such a service from community pharmacies and community pharmacists believed that this was an appropriate service to provide in their setting. Whilst most patients reported that they would be willing to pay for such a service, the actual cost, which was greater than £20, would be prohibitive to most and therefore alternative routes for funding would be required if this was to be provided as a routine service.

The large number of patients recruited means that we can estimate with 95 % confidence that the actual detection rate for such a service would be somewhere between 7 and 11.8 % which is relatively efficient when considering that only 1 % of the total population have the condition and would be found by population screening [3].

The response from the patient survey was limited with respondents not truly reflecting the demographics of those who had been recruited. Consequently their opinions may not be representative of all participants. Response rate could potentially be improved by asking participants to complete the survey within the pharmacy rather than taking it home, which was the method used here. Additionally asking patients how much they would be willing to pay for such a service may not reflect reality when the test can be provided for free by their general practitioner.

Whilst half of the community pharmacists involved in the study were interviewed there may be some self-selection bias with those less satisfied with the service being less willing to express their views in a face to face interview.

Many of the patients testing positive for coeliac disease were self-treating gastrointestinal symptoms and therefore the community pharmacy is an ideal location to place such a service. All patients receiving treatment for IBS will have been diagnosed with the condition by their GP and again these findings are in line with other studies regarding potential misdiagnosis of this condition [5]. In 2015 the UK NICE recommended that all patients are screened for coeliac disease prior to a confirmation of diagnosis of IBS [7] and if widely adopted this would increase detection rates.

Patients who provided feedback were uniformly positive about the setting, found the pharmacist able to answer their questions and would recommend the service to others. Individual patients commented on the professionalism exhibited by the pharmacists and on the usefulness of the information provided. Consequently this proof of concept study strongly suggests that if such a service were to be rolled out in a similar manner it would be acceptable to patients. With community pharmacists ideally located to monitor individuals once diagnosed with coeliac disease and trained to provide dietary advice, screening could be part of a more holistic service where the community pharmacist works collaboratively with the primary care team.

Participating community pharmacists were also positive regarding the experience, the training and the service because it enabled them to develop better customer relations, consider their needs more holistically and identify other services from which they may benefit. GPs were also reported to be receptive to the service. This may have been helped by the community pharmacies contacting them in advance of service provision.

With patients in this study reportedly unwilling to pay the actual cost of the service the question is whether the cost of screening high risk individuals in this manner would be offset by future reductions in health services cost. The development of a model to estimate the cost-effectiveness of this service from an NHS perspective is warranted.

The results of this service evaluation however can be used to remind community pharmacists that patients presenting for treatment of diarrhoea, IBS or non-specific gastrointestinal symptoms may actually have coeliac disease and therefore require referral.

This proof of concept study has shown that community pharmacies can be used to effectively screen for coeliac disease and, the process is acceptable to both patients and pharmacists. The cost-effectiveness of early detection of people with coeliac disease using community pharmacies however needs to be ascertained. Incorporating such screening within a more holistic community pharmacy based service including monitoring and advice may enhance the quality of care currently provided and potentially represent better value to commissioners.
